# Comparison of Endoscopy First and Laparoscopic Cholecystectomy First Strategies for Patients With Gallstone Disease and Intermediate Risk of Choledocholithiasis: Protocol for a Clinical Randomized Controlled Trial

**DOI:** 10.2196/18837

**Published:** 2021-02-04

**Authors:** Ausra Aleknaite, Gintaras Simutis, Juozas Stanaitis, Tomas Jucaitis, Mantas Drungilas, Jonas Valantinas, Kestutis Strupas

**Affiliations:** 1 Clinic of Gastroenterology, Nephrourology and Surgery Institute of Clinical Medicine Faculty of Medicine of Vilnius University Vilnius Lithuania; 2 Center of Hepatology, Gastroenterology and Dietetics Vilnius University Hospital Santaros Klinikos Vilnius Lithuania; 3 Center of Abdominal Surgery Vilnius University Hospital Santaros Klinikos Vilnius Lithuania

**Keywords:** choledocholithiasis, endoscopic ultrasound, intraoperative cholangiography, common bile duct stone, endoscopic retrograde cholangiopancreatography, laparoscopic cholecystectomy

## Abstract

**Background:**

The optimal approach for patients with gallbladder stones and intermediate risk of choledocholithiasis remains undetermined. The use of endoscopic retrograde cholangiopancreatography for diagnosis should be minimized as it carries considerable risk of postprocedural complications, and nowadays, less invasive and safer techniques are available.

**Objective:**

This study compares the two management strategies of endoscopic ultrasound before laparoscopic cholecystectomy and intraoperative cholangiography for patients with symptomatic cholecystolithiasis and intermediate risk of choledocholithiasis.

**Methods:**

This is a randomized, active-controlled, single-center clinical trial enrolling adult patients undergoing laparoscopic cholecystectomy for symptomatic gallbladder stones with intermediate risk of choledocholithiasis. The risk of choledocholithiasis is calculated using an original prognostic score (the Vilnius University Hospital Index). This index in a retrospective evaluation showed better prognostic performance than the score proposed by the American Society for Gastrointestinal Endoscopy in 2010. A total of 106 participants will be included and randomized into two groups. Evaluation of bile ducts using endoscopic ultrasound and endoscopic retrograde cholangiography on demand will be performed before laparoscopic cholecystectomy for one arm (“endoscopy first”). Intraoperative cholangiography during laparoscopic cholecystectomy and postoperative endoscopic retrograde cholangiopancreatography on demand will be performed in another arm (“cholecystectomy first”). Postoperative follow-up is 6 months. The primary endpoint is the length of hospital stay. The secondary endpoints are accuracy of the different management strategies, adverse events of the interventions, duct clearance and technical success of the interventions (intraoperative cholangiography, endoscopic ultrasound, and endoscopic retrograde cholangiography), and cost of treatment.

**Results:**

The trial protocol was approved by the Vilnius Regional Biomedical Research Ethics Committee in December 2017. Enrollment of patients was started in January 2018. As of June 2020, 66 patients have been enrolled.

**Conclusions:**

This trial is planned to determine the superior strategy for patients with intermediate risk of common bile duct stones and to define a simple and safe algorithm for managing choledocholithiasis.

**Trial Registration:**

ClinicalTrials.gov NCT03658863; https://clinicaltrials.gov/ct2/show/NCT03658863.

**International Registered Report Identifier (IRRID):**

DERR1-10.2196/18837

## Introduction

Gallbladder stones can be silent or symptomatic. This statement is also valid when speaking about choledocholithiasis, which involves stones situated in the common bile duct (CBD). About 10% to 18% of people undergoing cholecystectomy for gallstones have concomitant CBD stones [1]. Untreated choledocholithiasis can lead to acute biliary pancreatitis, acute ascending cholangitis, and secondary sclerosing cholangitis; thus, it is essential to diagnose and treat it on time. Endoscopic retrograde cholangiopancreatography (ERCP) became a prominent diagnostic method for CBD stones since its introduction to clinical practice in the 1970s [2]. Later on, it was agreed that use of ERCP for diagnostic reasons should be minimized and that it should not be used for first-line diagnostics as it carries a considerable risk (5% to 10%) of postprocedural complications [3]. It has been noticed that adverse events occur more often in patients with low risk of choledocholithiasis [4]. The possibility to avoid using ERCP for diagnostic purposes came with the introduction of new less invasive diagnostic procedures, such as magnetic resonance cholangiopancreatography (MRCP), endoscopic ultrasound (EUS), and intraoperative cholangiography (IOC) during cholecystectomy. Therefore, in the 2000s, a discussion about more careful patient selection for ERCP began, as it should be considered only in those with high probability of demand for therapeutic interventions (ie, stone removal), and patients with intermediate risk for choledocholithiasis should undergo additional investigation.

The most frequently used system to evaluate the risk of choledocholithiasis was proposed in 2010 by the American Society for Gastrointestinal Endoscopy (ASGE) [5]. It already stratifies the probability of CBD stones into low, intermediate, and high risk groups, and it suggests noninvasive investigations for the intermediate risk group, although its predictive value is not completely satisfying [6-10]. These results encourage the development of more accurate prognostic systems.

At the Center of Abdominal Surgery of Vilnius University Hospital Santaros Klinikos, an original prognostic index (Vilnius University Hospital Index [VUHI]) is being used for the prediction of choledocholithiasis risk before laparoscopic cholecystectomy (LC). Recently, we evaluated its accuracy and determined new threshold values for low, intermediate, and high risk groups [11]. The intermediate risk group (risk for choledocholithiasis of 25%-75%) would benefit from additional examination before ERCP. EUS and IOC are less invasive procedures with high accuracy for identifying CBD stones. These procedures will be applied for patients with intermediate risk of CBD stones and will help to decide if ERCP is indicated.

We aim to compare EUS as the first diagnostic biliary intervention to LC with IOC in patients with intermediate risk of choledocholithiasis in order to evaluate the accuracy, technical success, and safety of these two management strategies.

The hypothesis is that LC with IOC (“cholecystectomy first” strategy) will decrease both the length of hospital stay and morbidity by lessening the number of endoscopic investigations (EUS and ERCP) and thus the number of possible complications of ERCP, as well as decreasing the complications related to delayed cholecystectomy.

## Methods

### Recruitment

This study is a single-center, randomized, active-controlled trial comparing EUS and IOC for finding CBD stones in patients with intermediate risk of choledocholithiasis. Participants will be enrolled and the trial will be carried out at Vilnius University Hospital Santaros Klinikos, a tertiary referral center. All patients with planned LC due to gallstone disease will be evaluated for trial eligibility.

Ethical approval has been obtained from the Vilnius Regional Biomedical Research Ethics Committee (approval protocol number 158200-17-978-473).

The eligibility criteria are listed in Textbox 1 [12-14]. The trial will enroll patients with cholecystolithiasis aged 18 to 80 years, for whom LC is indicated and who have intermediate risk of choledocholithiasis. We will not include patients who are pregnant, morbidly obese (BMI >40), or severely ill (IV-VI class of the American Society of Anesthesiologists physical status classification, contraindications for general anesthesia or surgery). Additionally, patients with anastomosis in the upper gastrointestinal tract, known or suspected hepatitis of another origin (viral, toxic, etc), and other known cholestatic hepatopancreatobiliary disease will be excluded. We will rule out patients with known complications of gallstone disease, such as biliary pancreatitis, acute cholangitis, and acute cholecystitis (degree II-III, as defined in the Tokyo guidelines) [12,13].

**Eligibility criteria.**

**Inclusion Criteria**

- Age 18-80 years
- Symptomatic cholecystolithiasis (stones in the gallbladder seen on imaging studies and causing episodes of biliary colic)
- Intermediate risk of choledocholithiasis (Vilnius University Hospital Index 2.6-6.9 and one of the following predictors: dilated common bile duct >6 mm, elevated total bilirubin >21 µmol/L, or suspected stone in the common bile duct [CBD] on ultrasound)

**Exclusion Criteria**

- Acute cholangitis, as defined in the Tokyo guidelines 2013 [12]
- Moderately severe or severe biliary pancreatitis, as defined in the revised Atlanta classification [14]
- Acute cholecystitis (degree II-III), as defined in the Tokyo guidelines 2013 [13]
- Anastomosis in the upper gastrointestinal tract
- Known cholestatic hepatopancreatobiliary disease (primary biliary cholangitis, primary sclerosing cholangitis, secondary biliopathy, tumor of the head of the pancreas or major papilla, or benign or malignant CBD stricture)
- Known or suspected hepatitis (viral, toxic, alcoholic, etc) or liver cirrhosis
- Contraindications for general anesthesia or surgery
- IV-VI class of the American Society of Anesthesiologists physical status classification
- Morbid obesity (BMI > 40)
- Pregnancy
- Patient refusal to participate in the study

### Elimination From the Trial

Patients will be omitted from the trial if the situation changes to incompatible with the trial protocol. This can happen because of the following reasons: a neoplastic condition is found at the time of management; the general status of the patient worsens owing to other health issues not related to cholelithiasis (eg, myocardial infarction) and the patient needs urgent interventions not included in the trial protocol; and LC is converted to open cholecystectomy before IOC in the “cholecystectomy first” arm. Additionally, if the patient refuses to further participate in the trial, all the patient’s data are eliminated and further follow-up is not carried out. Informed consent will be obtained from all study participants.

### Randomization and Data Protection

Eligible patients who provide informed consent will be assigned to the groups “endoscopy first” or “cholecystectomy first” randomly, according to a premade sequence. The sequence is generated by a randomization website [15]. The sequence is created using block randomization of two elements A and B (“endoscopy first” and “cholecystectomy first”) in a ratio of 1:1. According to the sequence, sheets with group names are enclosed in opaque envelopes. Envelopes are numbered, and the envelope number is the patient number in the trial. When a new participant is enrolled, the topmost envelope is opened by one of the investigators and the participant is randomized into the specified group.

All collected data are coded, that is, every case receives an individual number. Only coded data will be employed for statistical analysis and publishing. Uncoded data are available only for researchers of the trial and, on special and reasonable request, for the coordination center for biomedical research of the institution and Biomedical Research Ethics Committee.

Data are processed and stored in an electronic database, and physical (“paper”) copies are stored at the trial center in accordance with procedures established by law.

### Procedure

The participants of the trial will undergo CBD evaluation depending on the group assignment. For the group “endoscopy first,” EUS is used to evaluate bile ducts. If stones are seen in the extrahepatic bile ducts, ERCP and CBD stone removal are performed during the same general endotracheal anesthesia. LC is performed after endoscopic procedures as soon as possible. In the group “cholecystectomy first,” LC with IOC is performed. If stones are found, postoperative ERCP with CBD stone removal is applied (during cholecystectomy if the CBD is completely blocked or as soon as possible).

EUS is performed with linear or radial Olympus ultrasound endoscopes. The CBD, pancreatic head, and adjacent structures are visualized from the duodenal bulb and descending duodenum. EUS is considered positive for a CBD stone when a constant hyperechogenic lesion with acoustic shadowing is seen in CBD projection.

ERCP procedures are performed by experienced endoscopists (each has more than 5 years of experience in ERCP and has done more than 500 procedures). Olympus side-viewing endoscopes (TJF-160VR) are used. Primary deep selective cannulation of the CBD is performed with a sphincterotome or cannula and guidewire technique. Diatrizoate (Urografin, Bayer) and iohexol (Omnipaque, GE Healthcare) are used as contrast media. Endoscopic sphincterotomy is performed over a guidewire technique with an Olympus pull-type sphincterotome. Papillary balloon dilation using a through-the-scope balloon catheter is applied when a stricture is indicated. Stones are removed using a retrieval balloon catheter and/or a Dormia basket. Complete clearance of the CBD is documented with a balloon catheter cholangiogram at the end of the procedure. ERCP is considered positive when a filling defect is seen in the cholangiogram and/or a stone is evacuated from the CBD. ERCP is considered unsuccessful when cannulation of bile ducts is technically impossible.

All patients will undergo a standard four-port LC (a 10-mm port at the umbilicus, a 10-mm port at the subxyphoid, a 5-mm port at the bottom of the gallbladder, and a 5-mm port at the right epigastrium). A 30-degree laparoscope is used for intra-abdominal visualization. After exposure and identification of the elements of the hepatocystic triangle, a small transverse cut is made in the cystic duct close to the gallbladder infundibulum with laparoscopic scissors. A 4-French cholangiogram catheter is placed in a 5-mm cholangiography fixation clamp and then inserted into the cystic duct. After verifying the absence of leakage at the catheter insertion site, contrast medium (Urografin) diluted in NaCl 0.9% solution (1:1 ratio) in a 20-mL syringe is injected under fluoroscopic vision (C-arm, Siemens GmbH). Cholangiograms are assessed by the operating surgeon and radiologist. IOC is considered positive when there is a filling defect or lack of contrast evacuation to the duodenum.

### Blinding

As both management strategies (endoscopic evaluation and intraoperative examination) differ in nature and postprocedure effect on the patient, complete blinding of participants is not possible. Before enrollment in the trial, the participant, treating clinician, and investigator will not know to which group the participant is assigned.

### Follow-Up

Participants are followed as treated inpatients after LC (short-term surveillance) and for 6 months after hospitalization (long-term surveillance). In the short-term surveillance period, postprocedural adverse events, signs of cholestasis, and need for repeated procedures are recorded. In the long-term surveillance period, participants are encouraged to contact the investigators if any symptoms of recurrent cholelithiasis are suspected. Participants will be contacted via phone or email 6 to 12 months later. Their health status will be evaluated using a questionnaire on the possible symptoms of choledocholithiasis (Multimedia Appendix 1). If any symptoms of possible gallstone disease are observed, the participant is invited for additional investigation (biochemical blood tests, transabdominal ultrasound, and MRCP on demand). All the enrollment, intervention, and surveillance procedures are listed in Multimedia Appendix 2.

### Statistical Analysis

The sample size was calculated in reference to collected data on the management of choledocholithiasis in the trial center Vilnius University Hospital Santaros Klinikos [11]. In our previous study, the mean treatment durations for different management strategy groups (LC-IOC first and ERCP first) were 5.37 and 7.13 days, with SDs of 2.5 and 2.8, respectively, and these findings were used to calculate the requested sample size. The program G*Power version 3.1.9.2 was used for calculations. It was calculated for a two-tailed *t* test for means of two independent groups. The significance level was selected to be .05, with a power of 0.8. The required sample size is 74 (37 valid participants in each of the two groups).

The endpoints in different management groups will be analyzed using the chi-square test or *t* test for independent means. Two-sided hypotheses are to be checked, and a *P* value <.05 will be considered statistically significant. If the distribution is nonnormal, a transformation, such as the logarithm or square root function, can be applied to obtain a normal distribution or nonparametric tests, such as the Mann-Whitney test, can be used. To evaluate the achieved power, a post-hoc power analysis calculation will be performed. As for the primary outcome, a difference of 2 days of hospital stay will be considered clinically meaningful.

### Outcomes

The primary endpoint is the length of hospital stay (duration from enrollment into the trial to discharge, in days).

The secondary endpoints are as follows:

1. Diagnostic accuracy of the different management strategies (proportion of correctly diagnosed [true positive and true negative] cases in the entire sample; time frame: 6 to 12 months).

2. Technical success of diagnostic and therapeutic biliary procedures (IOC, EUS, ERCP) (during the active treatment period). For IOC, successful cannulation and contrast media injection into the CBD are considered. For EUS, successful visualization of the CBD is considered. For ERCP, successful cannulation and contrast media injection into the CBD are considered. Successful CBD clearance is also considered.

3. Postoperative course and possible complications of treatment (time frame: up to 1 month). With regard to adverse events of endoscopic interventions and IOC, we consider (1) bleeding, hematemesis and/or melena, or hemoglobin drop >20 g/L; (2) perforation, evidence of air or luminal contents outside the gastrointestinal tract; (3) post-ERCP pancreatitis, new or worsening abdominal pain persisting for at least 24 h and requiring analgesics after ERCP in conjunction with an elevation in serum amylase or lipase levels greater than three times the normal upper limit [16,17]. We also consider assessment of the postoperative course by the Clavien-Dindo classification of surgical complications [18].

4. Cost of treatment (charges for diagnostic procedures, invasive procedures, surgery, and antibacterial treatment, if needed, as well as hospital charges).

## Results

The trial protocol was approved by the Vilnius Regional Biomedical Research Ethics Committee in December 2017. Enrollment of patients was started in January 2018. As of June 2020, 66 patients have been enrolled.

## Discussion

In the era of minimally invasive surgery and personalized medical care, the optimal cost-effective strategy for the management of patients with symptomatic gallstones and suspected choledocholithiasis has not been categorically defined yet.

The whole approach to patients with gallbladder stones consists of the following steps: evaluation of the probability of stones in the CBD, visualization, and evacuation of the stones when present along with treatment of cholecystolithiasis itself [19]. There are a few main clinical dilemmas in the management of choledocholithiasis, including which patients should be investigated for CBD stones and what is the optimal way to treat it (single-stage technique [LC with intraoperative CBD evaluation] or two-stage technique [preoperative ERCP followed by LC]).

First, it is essential to define the criteria for different risk groups. While the majority of recent trials evaluating the accuracy of choledocholithiasis prediction refer to ASGE guidelines, we performed an analysis of seven different studies on this prognostic system, and the predictive values of high-risk criteria were quite mediocre. The general sensitivity was 52.4%, specificity was 60.8%, positive-predictive value was 65.6%, negative-predictive value was 47.4%, and accuracy was 55.9% [11]. At the center of this trial, an original prognostic index (VUHI) has been used for the prediction of choledocholithiasis risk before LC since 1999. It is calculated by the following formula: VUHI = A / 30 + 0.4 × B, where A is the total bilirubin concentration (µmol/L) and B is the CBD diameter measured by ultrasound. The results of our previous study showed that the VUHI had comparable and, for some parameters, superior performance than the prognostic system of the ASGE guidelines [11]. The most modest measure was the specificity of VUHI (54%), while the sensitivity was 80.5%. This implied that earlier threshold of the index was kind of a weak spot in the evaluation system. The newly generated model for predicted probability of choledocholithiasis sets limits for the intermediate risk group, that is, it determines which patients should undergo additional noninterventional investigation. We chose thresholds for the intermediate risk group of 25% and 75% of the probability for CBD stones considering that the upper limit of 50% in the ASGE guidelines would still leave a certain number of patients for unnecessary ERCP. Latest European Association for the Study of the Liver (EASL) guidelines also state that patients with intermediate probability should undergo further evaluation with EUS or MRCP, but do not define what this intermediate probability is [20]. Meta-analyses showed that these two diagnostic procedures are quite comparable, but EUS has better diagnostic accuracy [21,22]. Just one trial comparing EUS and IOC was found in the PubMed database, and it showed better predictive values of IOC [23]. Considering that this study was performed 20 years ago and imaging technologies have advanced since then, it is worth comparing these two methods again. When comparing IOC with ERCP as a diagnostic procedure, a systematic review of 10 trials by Gurusamy et al showed slightly higher sensitivity for IOC with no difference in specificity [24].

All imaging methods are somehow operator dependent (or assessor dependent). EUS can have higher operator dependency as agreed by experts in the field because it requires not only evaluation of images but also proper positioning of the scope [25]. On the other hand, EUS is considered to be able to detect smaller CBD stones, which increases its value. Overall, this potentially is reflected in meta-analyses when evaluating not common specificity or sensitivity but the range in different studies. For example, Meeralam et al presented a pooled sensitivity and specificity of 0.97 (range 0.91-0.99) and 0.90 (range 0.83-0.94) for EUS and 0.87 (range 0.80-0.93) and 0.92 (range 0.87-0.96) for MRCP, respectively [21].

In terms of the level of “invasiveness” of these diagnostic methods, MRCP can be considered the least invasive; however, it has its own disadvantages, such as possible reaction to the contrast material and contraindications for the procedure (claustrophobia and ferromagnetic foreign objects). Additionally, EUS can be compared to conventional upper endoscopy, and the main possible complication is injury of the gastrointestinal tract wall with the scope, which is extremely rare. However, this procedure requires sedation or general anesthesia. IOC could seem to be the most invasive option because it is performed during operation, but it is just an additional step in an already ongoing surgery.

Determination of the best exploration method greatly depends on local expertise and availability of certain procedures. At our institution, availability of magnetic resonance imaging is limited because of the lack of equipment, so we decided to choose investigational procedures performed by surgeons and endoscopists themselves to compare.

The next step is to choose the optimal management strategy. In the aforementioned study, we assessed the effectiveness of different approaches (LC with IOC and ERCP “on demand” versus preoperative ERCP with sphincterotomy and necessary therapeutic interventions followed by LC). Some advantages in both strategies were found. There were less missed stones and false-positive cholangiographies in the ERCP first group. On the other hand, the LC-IOC group had less ERCP-related complications, and the mean length of hospital stay in this group was shorter, reflecting no need to wait for another procedure in most cases [11]. Barreras González et al also found these two strategies comparable in efficacy [26]. Moreover, meta-analyses of various different trials showed that there is no difference in the mortality, morbidity, retained stones, and failure rate between single-stage and two-stage choledocholithiasis management [1,27]. The main drawback of the preoperative ERCP plus LC strategy compared with various single-session approaches (intraoperative ERCP, LC with laparoscopic bile duct clearance, and open bile duct clearance) is higher time. Usually, there is a waiting period between the two procedures, which prolongs the duration of hospital stay and slightly increases the risk of developing recurrent biliary events and cholecystitis [20,28,29]. The reduced length of hospital stay (mean difference −3.01 days, 95% CI −3.51 to −2.50; *I²*=12%) was the only significant advantage of intraoperative ERCP found by a Cochrane systematic review when comparing single-stage and two-stage approaches in another way (laparoscopic endoscopic rendezvous versus preoperative endoscopic sphincterotomy) [30]. A recent meta-analysis by Ricci et al of four laparoscopic and endoscopic techniques for managing gallstone disease with biliary duct calculi showed that the safest and most successful approach is LC combined with intraoperative ERCP [31]. However, one of the biggest limitations of single-session ERCP and LC is difficult coordination of medical personnel, equipment, and location of the procedure [32,33]. Despite these restraints, a large survey of general surgeons in the United States showed that the majority of respondents preferred ERCP to laparoscopic CBD exploration for the management of choledocholithiasis if CBD stones were diagnosed preoperatively or intraoperatively [34]. IOC followed by laparoscopic CBD exploration is another possible single-stage strategy. This method appeared to be the safest for avoiding bleeding, took the shortest operative time, and was the least costly in the review by Ricci et al [31]. Unfortunately, this method is not usually applied in our institution, so we decided not to involve it in the trial owing to lack of local expertise.

CBD clearance was found to be alleviated by flushing saline after anterograde balloon dilatation of the Oddi sphincter or after glucagon injection, but these methods are described in singular trials and confirmatory studies are needed [35-37].

This trial is aimed to clarify which one of the two strategies (preoperative EUS or LC with IOC) is the optimal solution for patients with intermediate risk of CBD stones. We intend to compare various aspects of the two approaches of choledocholithiasis management, ranging from accuracy to cost and time efficiency. The thresholds of different risk groups according to the VUHI (original prognostic index) will also be verified prospectively. The index was designed to evaluate the risk for CBD stones in patients with gallbladder stones and an intact gallbladder [38]. Symptomatic cholelithiasis is the main inclusion criterion because it is a major indication for cholecystectomy. We will include adult subjects under 80 years of age as the CBD diameter tends to increase with age [39]. Individuals with other diseases that can cause cholestasis or abnormal liver function test results will be excluded. The diseases include parenchymal disease and mechanical obstruction (from primary sclerosing cholangitis to tumor of the pancreas), as well as biliary pancreatitis, which has been found to not be associated with the risk of choledocholithiasis [10,40,41]. Additionally, we chose to exclude patients with severe acute cholecystitis or cholangitis, as these cases must receive immediate intervention. Finally, patients with absolute or conditional contraindications or burdensome factors for surgery or ERCP (eg, morbid obesity, Billroth II type resection, and severe general condition) are ruled out. We presume that LC with IOC could be the preferred management strategy because of saved time compared with a two-stage strategy, so the study is planned as a superiority trial. We chose to designate hospital stay of 2 days as the minimal important difference between the two groups because it causes a considerable increase in management cost and is barely influenced by nonmedical reasons, which can happen when the difference is chosen to be 1 day. If the difference in inpatient treatment duration is not statistically significant, there are yet other outcomes to be evaluated to compare these two strategies. Overall, this trial is planned to define a simple and safe algorithm for managing choledocholithiasis.

## Appendix

Multimedia Appendix 1 Possible symptoms of choledocholithiasis.

Multimedia Appendix 2 Enrollment, intervention, and surveillance procedures.

## Abbreviations

ASGE: American Society for Gastrointestinal Endoscopy
CBD: common bile duct
ERCP: endoscopic retrograde cholangiopancreatography
EUS: endoscopic ultrasound
IOC: intraoperative cholangiography
LC: laparoscopic cholecystectomy
MRCP: magnetic resonance cholangiopancreatography
VUHI: Vilnius University Hospital Index
