# Supplementary material for: Comparison of Endoscopy First and Laparoscopic Cholecystectomy First Strategies for Patients With Gallstone Disease and Intermediate Risk of Choledocholithiasis: Protocol for a Clinical Randomized Controlled Trial
Source: JMIR Res Protoc. 2021 Feb 4;10(2):e18837. doi: 10.2196/18837 (PMC7892280; doi:10.2196/18837)
Supplement: Multimedia Appendix 1 [file resprot_v10i2e18837_app1.docx]

**Questionnaire 1. Symptoms of possible choledocholithiasis.**

You had a gallbladder removal operation performed in Santaros Klinikos 6 months ago. You have consented to participate in the clinical study on risk of bile duct stones. Please answer to following questions on your health status after the operation.

1. Have you had any pain in the upper right part of your abdomen or in other areas? If yes, please specify.
2. Have you become jaundiced (have you noticed that your skin or eyes turned yellow)?
3. Have you had fever without any clear reason (e.g., common cold)?
4. Have you had doctor appointment for any of those reasons?
5. If yes, what investigations were performed and what diagnosis was stated?
6. If you had any of those symptoms, would you agree to arrive at Santaros Klinikos for additional investigation – abdominal ultrasound and blood tests?
