# Supplementary material for: Comparison of Endoscopy First and Laparoscopic Cholecystectomy First Strategies for Patients With Gallstone Disease and Intermediate Risk of Choledocholithiasis: Protocol for a Clinical Randomized Controlled Trial
Source: JMIR Res Protoc. 2021 Feb 4;10(2):e18837. doi: 10.2196/18837 (PMC7892280; doi:10.2196/18837)
Supplement: Multimedia Appendix 2 [file resprot_v10i2e18837_app2.docx]

Table 1. Enrolment, interventions and surveillance procedures.

|  | Study period | | | | |
| --- | --- | --- | --- | --- | --- |
|  | Enrolment | Allocation | Post-allocation | | |
|  |  |  | Management | Short term surveillance  (inpatient) | Long term surveillance  (6 months) |
| **Recruitment***:* |  |  |  |  |  |
| Eligibility screen | x |  |  |  |  |
| Informed consent | x |  |  |  |  |
| Randomization |  | x |  |  |  |
| Management strategy*:* |  |  |  |  |  |
| “Endoscopy first” |  |  | x |  |  |
| “Cholecystectomy first” |  |  | x |  |  |
| Assessments: |  |  |  |  |  |
| Bilirubin concentration | x |  |  | on demand | on demand |
| Ultrasound | x |  |  | on demand | on demand |
| Surgical records |  |  | x | x |  |
| Postoperative records |  |  | x | x |  |
| Interview with patient |  |  |  |  | x |
